# Soil respiration of four forests along elevation gradient in northern subtropical China

**DOI:** 10.1002/ece3.5762

**Published:** 2019-11-06

**Authors:** Mingzhe Ma, Zhenhua Zang, Zongqiang Xie, Quansheng Chen, Wenting Xu, Changming Zhao, Guozhen shen

**Affiliations:** ^1^ State Key Laboratory of Vegetation and Environmental Change Institute of Botany Chinese Academy of Sciences Beijing China; ^2^ University of Chinese Academy of Sciences Beijing China; ^3^ The Key Laboratory for Silviculture and Conservation of Ministry of Education Beijing Forestry University Beijing China

**Keywords:** autotrophic respiration, heterotrophic respiration, intersection effect, *Q*_10_, soil moisture, soil temperature

## Abstract

**Background and aims:**

Soil respiration is the second‐largest terrestrial carbon (C) flux, and soil temperature and soil moisture are the main drivers of temporal variation in soil respiration and its components. Here, we quantified the contribution of soil temperature, soil moisture, and their intersection on the variation in soil respiration and its components of the evergreen broad‐leaved forests (EBF), mixed evergreen and deciduous broad‐leaved forests (MF), deciduous broad‐leaved forests (DBF), and subalpine coniferous forests (CF) along an elevation gradient.

**Methods:**

We measured soil respiration of four types of forests along the elevation gradient in Shennongjia, Hubei China based on the trenching experiments. We parameterized the relationships between soil respiration and soil temperature, soil moisture, and quantified the intersection of temperature and moisture on soil respiration and its components.

**Results:**

Total soil respiration (*R*_S_), heterotrophic respiration (*R*_H_), and autotrophic respiration (*R*_A_) were significantly correlated with soil temperature in all four forests. The *Q*_10_ value of soil respiration significantly differed among the four types of forest, and the *Q*_10_ was 3.06 for EBF, 3.75 for MF, 4.05 for DBF, and 4.49 for CF, respectively. The soil temperature explained 62%–81% of the variation in respiration, while soil temperature and soil moisture together explained 91%–97% of soil respiration variation for the four types of forests. The variation from the intersection of soil temperature and moisture were 12.1%–25.0% in R_S_, 1.0%–7.0% in *R*_H,_ and 17.1%–19.6% in *R*_A,_ respectively.

**Conclusions:**

Our results show that the temperature sensitivity (*Q*_10_) of soil respiration increased with elevation. The intersection between soil temperature and soil moisture had strong effects on soil respiration, especially in *R*_H_. We demonstrated that the intersection effects between soil temperature and soil moisture on soil respiration were essential to understand the response of soil respiration and its components to climate change.

## Graphical abstract

Partitioning of soil respiration is helpful for understanding the response to climate change. The interactive effects of soil temperature and soil moisture showed different between *R*_A_ and *R*_H_. *R*_H_ explained by the interaction increased with elevation. Elevation effect on each component should be taken into consideration.

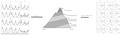

## 1 INTRODUCTION

Soil carbon represent 80% of the global terrestrial ecosystem carbon stock, 2–3 times more than the terrestrial vegetation carbon pool (500–600 Gt), and twice the atmospheric carbon pool (750 Gt; Bond‐Lamberty & Thomson, 2010; Hashimoto et al., 2015; Schlesinger, 1990). Forest soil carbon maintains 86% of the global vegetation carbon pool and 73% of the soil carbon pool (Deluca & Boisvenue, 2012; Dixon et al., 1994; Tans, Fung, & Takahashi, 1990).

Soil CO_2_ efflux, commonly referred to as soil respiration (*R*_S_), is the primary path by which *C* fixed by land plants returns to the atmosphere (Barba et al., 2018). Estimated at approximately 75 × 10^15^ gC/year, this large natural flux is likely to increase due to changes in the earth's condition (Bond‐Lamberty & Thomson, 2010). The total global emission of CO_2_ from soils is recognized as one of the largest fluxes in the global carbon cycle, and small changes in the magnitude of soil respiration could have a large effect on the concentration of CO_2_ in the atmosphere (Schlesinger & Andrews, 2000).

Large uncertainty exists in soil respiration estimation because soil respiration is regulated by multiple abiotic and biotic factors, such as soil temperature, moisture, nutrient availability, and plant productivity (Chen, Xu, Yu, & Ding, 2017). Furthermore, soil respiration consists of two main components, heterotrophic (*R*_H_) and autotrophic respiration (*R*_A_), which respond differently to changes in influencing factors (Ryan & Law, 2005). R_H_ mainly comes from free‐living soil microorganisms that subsisted by decomposition of soil organic matter and organic matter in litter layer (Högberg et al., 2001; Scott‐Denton, Rosenstiel, & Monson, 2006). *R*_A_ mainly comes from roots, mycorrhizae, and other microorganisms that are in obligate associations with living roots and the organic exudates provided by aboveground parts of the plant through photosynthates. (Bond‐Lamberty & Thomson, 2010). Thus, partitioning *R*_S_ into its components and assessing their responses to soil temperature and moisture are essential to improve our mechanistic knowledge and model prediction of R_S_ under various environmental conditions and management practices (Chen et al., 2017; Hopkins et al., 2013; Subke, Inglima, & Cotrufo, 2006).

Soil temperature and moisture are well known to be dominant environmental controls on respiration rate due to their direct effects that alter the activities of soil microbes and plant roots, and indirect effects through changing substrate supply and plant growth (Hanson et al., 2000; Ise & Moorcroft, 2006; Schimel et al., 1994). Variation in soil temperature and moisture can account for most of the seasonal and diel variation in soil CO_2_ efflux (Davidson, Belk, & Boone, 1998). Rising temperatures stimulated soil respiration by accelerating rates of C cycling via autotrophic respiration and heterotrophic decomposition of organic matter (Bond‐Lamberty & Thomson, 2010; Melillo et al, 2011). Soil moisture is a main driver of net primary productivity and thus strongly affects carbon inputs as well as the decomposition of litter and soil organic matter, and hence, heterotrophic respiration and carbon outputs (Moyano, Manzoni, & Chenu, 2013). To date, there is mounting evidence that the temperature sensitivity of respiration declines with increasing temperature and decreasing soil moisture (Flanagan & Johnson, 2005; Janssens & Pilegaard, 2003; Kirschbaum, 1995; Reichstein et al., 2002).

However, previous analyses have focused on the effects of either temperature or soil moisture on forest soil respiration, little partitioned the effect of the intersections of temperature and soil moisture on soil respiration and its components (Taylor et al., 2017). The limited understanding of the intersection effects constrains our ability to predict ecosystem carbon fluxes under future climate regimes (Flanagan & Johnson, 2005).

The altitude gradient shows climate gradients under similar geographic scales, enriches different vegetation types, and concentrates many bioecological processes (Malhi et al., 2010). Montane elevation transects also make excellent natural laboratories for understanding the intersection of soil temperature and moisture on soil respiration (Körner, 2007; Malhi et al., 2010; Sundqvist, Sanders, & Wardle, 2013). The elevation gradient of mountains in Shennongjia condenses four types of forests, including evergreen broad‐leaved forests (EBF), mixed evergreen and deciduous broad‐leaved forests (MF), deciduous broad‐leaved forests (DBF), and subalpine coniferous forests (CF) in a small horizontal distance (Ma et al., 2017). Here, we explored the effects of soil temperature and soil moisture and their intersection effects on *R*_S_, *R*_A_, and *R*_H_ of the four types of forest along the elevation gradient in Shennongjia, northern China. Our objectives were as follows: (a) to partition soil respiration into autotrophic respiration and heterotrophic respiration of four types of forest along the elevation gradient, (b) to examine the responses of soil respiration and its components of four types of forest along the elevation gradient to soil temperature and moisture, and (c) to quantify the intersection effect of soil temperature and moisture on soil respiration and its components of four types of forest along the elevation gradient.

## 2 MATERIALS AND METHODS

### 2.1 Site description

The research was conducted at National Field Station for Forest Ecosystem of Shennongjia in the eastern Daba Mountains, Hubei province, China (109°56′–110°58′E, 31°15′–31°57′N). The field station has a typical north subtropical monsoon climate, with an annual average precipitation of 1,306–1,722 mm, of which nearly 80% rain falls in the wet season (from April to September) and 20% in the dry season (from October to March). The mean annual temperature is 10.6°C. Less affected by the Quaternary glaciation, Shenongjia preserved the intact vegetation zonation in the Oriental Deciduous Forest Biogeographical Province (Udvardy, 1975), including evergreen broad‐leaved forests (EBF), mixed evergreen and deciduous broad‐leaved forests (MF), deciduous broad‐leaved forests (DBF), and subalpine coniferous forests (CF) along the elevation gradient, which ranges from 420 m to 3,100 m above sea level.

### 2.2 Experimental design

We established experimental sites in the four types of forest along the elevation gradient in Shennongjia, Hubei, China (Tables 1 and 2). Within each forest type, we established three plots (25 × 25 m). Each plot was divided into twenty‐five blocks (5 × 5 m), and one subplot (100 × 100 cm) was positioned in each block. We positioned the subplot in the center of the block. The minimum distances between trenched plots and nontrenched plots were 4 m (the trenched neighbored the nontrenched block), and the maximum distances were 9 m (a blank block between the trenched and nontrenched block). In September and October 2008, we randomly chose sixteen subplots (100 × 100 cm) from twenty‐five subplots (100 × 100 cm) for each plot, so there were sixteen subplots for the measurement in each plot. We dug trenches along the edges of eight subplots, with depth to the bedrock and width of 10 cm, and the rest eight subplots were untrenched subplots. Trenches were lined with hard sponge, refilled and packed carefully with the soil (We found no corrosion or decomposition of hard sponge in the pre‐experiment, in the whole experimental process and the recovery after the end of the experiment). Then, we carefully removed all aboveground vegetation with minimal soil disturbances and kept the trenched plots free of live vegetation throughout the study period.

**Table 1:** Site characteristics of four types of forest along the elevational gradient in Shennongjia, Hubei, China

Location	Elevation (m)	Slope	Precipitation (mm)	Mean diameter at breast height (cm)	Dominant species	Forest type
31°28′N 110°18′E	2,570	22.0°	1,100	24.82	*Abies fargesii*, *Abies chensiensis*	CF
31°18′N 110°30′E	1,970	19.0°	1,050	17.59	*Quercus aliena* var. *cutiserrata*, *Cronus japonica* var. *hinensis*	DBF
31°19′N 110°29′E	1,670	21.0°	1,200	13.34	*Fagus engleriana*, *Cyclobalanopsis glauca*	MF
31°21′N 110°30′E	780	41.5°	850	15.85	*Lindera strychnifolia* var. *hemsleyana*, *Phoebe zhennanyichang*, *Cyclobalanopsis glauca*	EBF

**Table 2:** Soil properties of four types of forest along the elevational gradient in Shennongjia, Hubei

Parameters	Forest type			
	EBF	MF	DBF	CF
Soil type	Cambosols	Argosols	Argosols	Argosols
Soil texture	Clay	Silt Loam	Loam	Sandy Loam
pH (H_2_O)	6.8 ± 0.3	6.0 ± 0.3	5.2 ± 0.2	4.8 ± 0.2
Organic C (%)	4.06 ± 1.05	4.01 ± 0.37	1.53 ± 0.38	2.09 ± 0.45
Total C (%)	4.27 ± 1.29	4.01 ± 0.37	1.75 ± 0.67	2.09 ± 0.45
Total N (%)	0.38 ± 0.09	0.37 ± 0.03	0.17 ± 0.04	0.21 ± 0.04
Total P (mg/g)	0.54 ± 0.11	0.76 ± 0.04	0.42 ± 0.13	0.61 ± 0.07

Note

Values are mean ± *SE*.

Soil type reference *Chinese Soil Taxonomy (CST1999)*. Soil texture reference *USDA's soil texture classification*.

### 2.3 Measurements of soil respiration, soil temperature, and water content

A PVC collar (20.3 cm in diameter and 10 cm in height) was inserted into the soil in each trenched and untrenched (192 trenched and untrenched subplots in total) subplot with depth of 2.5 cm at each sampling point approximately 2 weeks before the first measurement. Small litter was left in the collar, and large items (fallen wood, rock block, etc.) were removed. All collars were left at the site for the entire study period.

We measured the soil surface CO_2_ fluxes from 2009 to 2011. Soil respiration data from the 192 PVC collars were measured once every 15 days over the whole period of growth season from May to November, and once every 30 days over the whole period of nongrowth season from December to March. The suitable diurnal measurement time was determined based on the preliminary experiment in 2008 (continuous measurement of soil respiration). According to the results of the preliminary experiment, we chose 8:30–12:00 a.m. as the most suitable time of the day for measurements, and then we measured the CO_2_ flux at 8:30–12:00 a.m. from 2009 to 2011. We measured the CO_2_ flux by the automated soil CO_2_ flux system (Li‐8100; LI‐COR) equipped with a portable chamber.

We measured temporal soil temperature (*T*, °C) and soil water content (SWC, g/100 g) near each collar at the same time assoil respiration measurements. Soil temperature was measured at a depth of 10 cm using a handle thermocouple probe, while the soil volumetric water content was measured at 0–10 cm depth, using a moisture meter equipped with the Li‐8100. Similarly, we recorded the soil temperature and moisture near each collar at 2‐min intervals throughout the entire study period. Soil temperature was measured at 10 cm depth by a thermos‐recorders, and soil moisture was measured at 10 cm by a soil moisture sensor (HOBO). We aimed to calibrate and find outliers to reduce measurement errors.

### 2.4 *Estimation of R*_H_ *and R*_A_

We cut off the carbon input from the roots outside the treatment plots by trenching. Because the remaining roots in the trenched plot was likely to increase the substrate supply for microbial respiration and thus raise CO_2_ flux from the trenched plots (Lee, Nakane, Nakatsubo, & Koizumi, 2003). So, we measured the root decomposition.

We sampled the roots from five destructive plots (1 m × 1 m) neighboring the measured plots randomly depth to bedrock in each type of forest in August 2008. Then, we collected the roots by washing and brushing the soil from the destructive subplots with deionized water and separated the roots into fine roots (0–2 mm), medium roots (2–5 mm), and coarse roots (5–10 mm). We air‐dried the fine roots to constant mass, and then weighed the roots (0.001 g).

We measured the decomposition of the fine roots, medium roots, and coarse roots through litterbag method (Lee et al., 2003). We filled each litterbag (20 × 20 cm nylon mesh bags of 1 mm mesh) with 5.00 ± 0.01 g of air‐dried roots (We washed and dried the roots of each block, then mixed fine, medium, and coarse roots together, and then sampled 5 g from the mixture for litterbag, and the proportion of each root type was same with the roots before separation according to diameter classification of the roots) and placed the litterbag horizontally in the soil depth of 10–20 cm in each subplot in 2008. We retrieved five litterbags from each plot in May, July, September, and November in 2009, and March, May, and July in 2010, so 35 litterbags were retrieved in each plot. We then removed the soil particles and other extraneous materials of the root samples and oven‐dried the roots to constant mass and weighed the roots.

We analyzed the root decomposition by Olson's (1963) standard exponential decay function *X*/*X*_0_ = *ae* ^−^ *^kt^*, where *X*/*X*_0_ is the fraction of initial mass remaining (*X* = root mass at time *t* and *X*_0_ = initial mass), *t* is time (year^−1^), and *k* is the relative loss rate of root mass (the slope of the linear regression fit for roots of each class; *a* = intercept).

We removed the CO_2_ fluxes released from root decomposition (*R*_D_) when we calculated *R*_H_. Root decomposition has a direct relationship with the relative loss rate constant (*k*). We used 2/3 as the decomposition rate (Lee et al., 2003). We calculated the root decomposition rates (*v*) by the equation:

v=0.64k.

And then, we calculated the CO_2_ fluxes released from the residual root decomposition of each size class (*R*_d_; g C m^−2^ day^−1^) at a given time *t* by

$$R_{d}=B_{r}\left(ae^{-v(t-1)}-ae^{-vt}\right).$$

where *R*_D_ was the sum of the *R*_d_ values of each size class (*R*_D_ = Σ*R*_d_), and *B*_r_ was abbreviation of the root biomass.

We calculated *R*_A_ by the following equation:

$$R_{A}=R_{\text{untrench}}-\left(R_{\text{trench}}-R_{D}\right)$$

where *R*_untrench_ was soil respiration rate in the untrenched plot, *R*_trench_ was soil respiration rate in the trenched plot, so we calculated *R*_H_ as

$$R_{H}=R_{\text{untrench}}-R_{A}$$

The total *R*_S_ was soil respiration rate in the untrenched plot:

$$R_{S}=R_{\text{untrench}}=R_{A}+R_{H}$$

The annual *R*_S_ rate was the average of the rate of the whole year respiration.

### 2.5 Temperature sensitivity and soil water content sensitivity

We estimated *Q*_10_ values by the first‐order exponential equation from Van't Hoff, the most commonly used equation to express the temperature sensitivity of soil respiration (Davidson, Janssens, & Luo, 2006). Where *T* was soil temperature at 10 cm depth, and *a*, *b* were fitted parameters. We calculated the temperature sensitivity (*Q*_10_) of soil respiration by the following equation:

$$R_{S}=ae^{\mathrm{bT}}$$

$$Q_{10}=e^{10b}.$$

The relationship between *R*_S_ and soil moisture contents was fitted by a linear function. The *W*_slope_ was the soil water content sensitivity of *R*_S_

$$R_{S}=W_{\text{slope}}\text{SWC}+b.$$

### 2.6 Soil respiration partition

**Figure 1:**
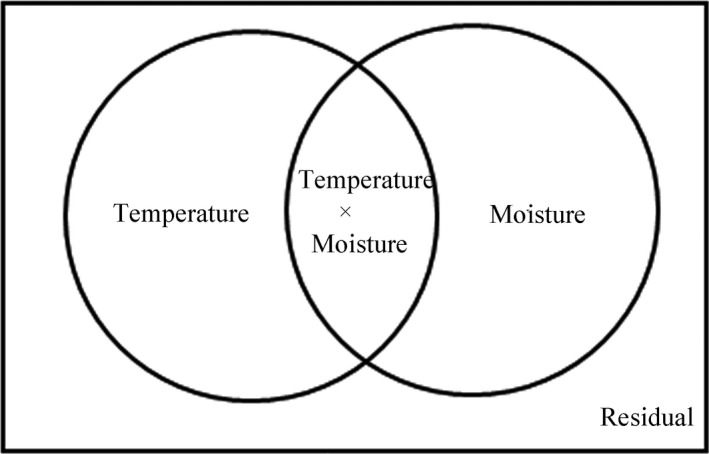
The partition of variations in *R*_S_, *R*_H_, and *R*_A_

We partitioned the variations of *R*_S_, *R*_H_, and *R*_A_ into [*a*], [*b*], [*c*], and [*d*] (Figure 1). So [*a* + *b*] represent the soil respiration variation derived from the soil temperature, [*b* + *c*] represented the soil respiration variation derived from the soil moisture, while [*b*] represented the soil respiration variation derived from the intersection of soil temperature and soil moisture, and [*d*] represented the residual variation of the soil respiration derived from some other factors

The exponential equation (*R*_S_ = a*exp(b*T)) was widely used to express the relationship between soil respiration and soil temperature (*T*; Boone, Nadelhoffer, Canary, & Kaye, 1998; Wang, Yang, & Zhang, 2010), while the linear function (*R*_S_ = a* *M* + *b*) was widely used to express the regression relationship of soil moisture (*M*) with soil respiration (Raich & Schlesinger, 1992; Wu, Dijkstra, Koch, Peñuelas, & Hungate, 2011). By computing regression *y*(*R*_S_) against temperature, moisture, and multiple regression against temperature and moisture together, we got the regression values: [*a* + *b*], [*b* + *c*], [*a* + *b* + *c*].

We calculated “*a*” by [*a*] = [*a* + *b* + *c*] − [*b* + *c*]; likewise, fraction [*c*] was computed by [*c*] = [*a* + *b* + *c*] − [*a* + *b*]; [*b*] was also obtained by [*b*] = [*a* + *b*] + [*b* + *c*] − [*a* + *b* + *c*] or [*b*] = [*a* + *b*] − [*a*] or [*b*] = [*b* + *c*] − [*c*], and [*d*] = 1 − [*a* + *b* + *c*].

### 2.7 Statistical analysis

We investigated the relationships between the *R*_S_, *R*_A_, and *R*_H_ with the soil temperature using exponential regression analysis, respectively, and the relationships between the *R*_S_, *R*_A_, and *R*_H_ with the soil water content using linear regression analysis, respectively. We analyzed the relationship between *Q*_10_ with the parameters of elevation using linear regression analysis. The relationships in Figure 3 were based on Pearson correlation analysis. We explored the differences of *R*_S_, *R*_H_, and *R*_A_ among the four types of forest by repeated measures ANOVA (*α* = 0.05). We investigated the effects of the trenching treatment on soil respiration rate with a paired *t* test. We compared the relationship between respiration rate and soil temperature with one‐way ANOVA (*α* = 0.05). We conducted the analyses with SAS software (SAS Institute Inc.).

## 3 RESULTS

### 3.1 *Patterns of R*_S_ *, R*_H_ *, and R*_A_

Temporal variations of soil respiration in the four types of forest along the elevation showed a distinct “bell‐shape” trend. There was significant variation in annual flux of soil respiration among the four types of forest along the elevation gradient in Shennongjia. *R*_S_ and *R*_H_ of CF was the lowest among the four types of forest (Table 3), while *R*_A_ of CF was significantly lower than DBF and MF.

**Table 3:** Total *R*_S_, *R*_A_, and *R*_H_ of four types of forest along the elevation gradient in Shennongjia, Hubei, China

Forest type	*R*_s_	*R*_H_	*R*_A_
CF	1.35 ± 0.05^a^	0.81 ± 0.04^a^	0.54 ± 0.09^b^
DBF	1.72 ± 0.10^b^	1.17 ± 0.10^b^	0.56 ± 0.20^b^
MF	1.79 ± 0.06^b^	1.12 ± 0.09^b^	0.67 ± 0.15^a^
EBF	1.63 ± 0.06^b^	1.13 ± 0.05^b^	0.50 ± 0.11^c^

Note

Values are mean ± *SE* (μmol CO_2_ m^–2^ s^–1^). The superscript letters indicated the significant differences between forest types (*p* = .05, repeated measures ANOVA).

**Figure 2:**
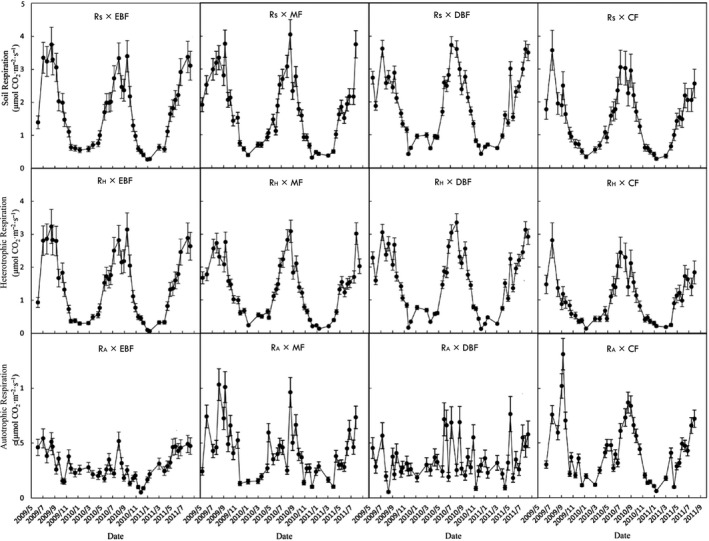
Seasonal pattern of *R*_S_, *R*_H_, and *R*_A_ of four types of forest along the elevation gradient in Shennongjia, Hubei, China

Soil respiration flux in summer and autumn was significantly higher than in winter and spring (*p* < .001) for four types of forests (Figure 2). In which, annual *R*_S_ rate of MF was the highest among the four types of forest. The annual *R*_H_ rate of DBF was the highest among the four types of forests (Table 3, Figure 2). *R*_A_ in MF and R_A_ in CF were not significantly different (*p* < .05), and the difference of *R*_A_ among EBF, MF (CF), and DBF were significant (*p* < .05; Table 3).

### 3.2 The sensitivity of *R*_S_, *R*_H_, and *R*_A_ to soil temperature and to soil moisture

**Figure 3:**
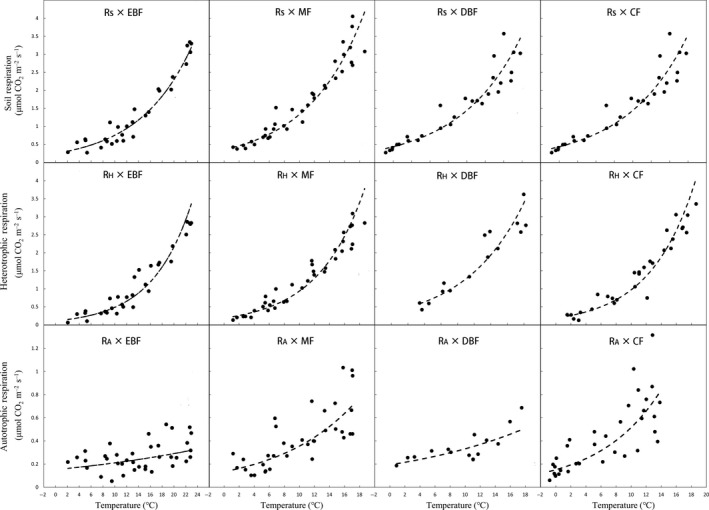
Relationship between *R*_S_, *R*_H_, and *R*_A_ and soil temperature of four types of forest along the elevation gradient in Shennongjia, Hubei (The *p* value was less .001 in *R*_S_ and *R*_H_ and less .01 in *R*_A_.)

*R*_S_, *R*_H_, and *R*_A_ were significantly correlated with soil temperature in four types of forests (Figure 3, Table 4). The *Q*_10_ of *R*_S_ and *R*_H_ increased with the elevation increase (Table 4), except the mixed forest. For MF, the temperature sensitivity was higher than DBF. The soil moisture sensitivity of *R*_S_ and *R*_A_ significantly increased with the elevation (Table 5).

**Table 4:** Regression between the soil temperature and the *R*_S_, *R*_H_, and *R*_A_ of four types of forest along the elevation gradient in Shennongjia, Hubei, China

Respiration	Forest type	*a*	*R* ^2^	*b*	*Q*_10_
*R*_S_	CF	0.43	0.94	0.15	4.49^a^
	DBF	0.36	0.91	0.14	4.05^b^
	MF	0.35	0.94	0.13	3.75^c^
	EBF	0.25	0.88	0.11	3.06^d^
*R*_H_	CF	0.26	0.94	0.17	5.48^a^
	DBF	0.18	0.90	0.17	5.27^b^
	MF	0.19	0.92	0.16	5.02^b^
	EBF	0.11	0.86	0.15	4.47^c^
*R*_A_	CF	0.15	0.70	0.12	3.40^a^
	DBF	0.14	0.53	0.09	2.39^b^
	MF	0.19	0.58	0.05	1.71^c^
	EBF	0.15	0.15	0.03	1.38^d^

Note

The superscript letters of *Q*_10_ indicated the significant differences (*p* < .05, One‐Way ANOVA). The *p* value was less .001 in *R*_S_ and *R*_H_ and less .01 in *R*_A_. Function was *R*_S_ = *ae^bT^*.

**Table 5:** Variation partition of *R*_S_, *R*_H_, and *R*_A_ of four typical forests along the elevation gradient in Shennongjia, Hubei, China

Respiration	Forest type	[*a*]	[*b*]	[*c*]	[*a* + *b* + *c*]	[*a* + *b*]	[*b* + *c*]
*R*_S_	CF	0.81	0.12	0.01	0.95	0.93	0.13
	DBF	0.79	0.11	0.01	0.91	0.90	0.12
	MF	0.80	0.12	0.01	0.93	0.92	0.14
	EBF	0.63	0.25	0.06	0.94	0.88	0.31
*R*_H_	CF	0.86	0.07	0.04	0.97	0.93	0.11
	DBF	0.90	0.01	0.03	0.93	0.91	0.04
	MF	0.87	0.05	0.02	0.94	0.92	0.07
	EBF	0.85	0.01	0.01	0.87	0.86	0.02
*R*_A_	CF	0.47	0.17	0.03	0.67	0.64	0.20
	DBF	0.38	0.17	0.01	0.56	0.54	0.18
	MF	0.34	0.17	0.01	0.53	0.52	0.19
	EBF	0.12	0.20	0.003	0.32	0.32	0.20

### 3.3 Soil respiration variance partitioning of soil temperature and soil moisture

The trenched treatment did not significantly change the soil temperature and soil moisture of the four types of forests along the elevation gradients (Table 6). The variation of *R*_S_, *R*_H_, and *R*_A_ explained by soil temperature and soil moisture was 91%–95%, 87%–97%, and 32%–67%, respectively (Table 5). Totally, the soil temperature accounted for 87.9%–93.5%, 85.9%–93.3%, and 31.7%–64.1% of the variation in R_S_, R_H_, and R_A_, respectively, and soil moisture accounted for 12.0%–31.3%, 2.1%–10.9%, and 18.3%–19.9% of the variation in *R*_S_, *R*_H_, and *R*_A_, respectively (Table 5).The variation of *R*_S_, *R*_H_, and *R*_A_ explained only by soil temperature was 84.1%–89.7%, 63.0%–81.4%, and 12.1%–47.0%, respectively (Table 5), and the variation increased with the elevation increase. Similarly, the variation of *R*_S_, *R*_H_, and *R*_A_ explained only by soil moisture were 1.1%–6.3%, 1.1%–4.0%, and 0.3%–2.6%, respectively (Table 5). The variation of *R*_S_, *R*_H_, and *R*_A_ explained by the intersection of soil temperature and moisture were 12.1%–25.0%, 1.0%–7.0%, and 17.1%–19.6%, respectively, and the variation of *R*_S_ and *R*_A_ explained by the intersection of soil temperature and moisture decreased with the elevation increase, while the variation of *R*_H_ explained by the intersection between soil temperature and moisture showed no significant correlation with the elevation (Table 5). The results indicated that *R*_S_, *R*_H_, and *R*_A_ of the four types of forest along the elevation gradient in Shennongjia was mainly dominated by soil temperature, but that soil moisture also had an important influence on soil respiration.

**Table 6:** Mean soil temperature and soil moisture in controlled and the trenched plots of four types of forest along the elevation gradient in Shennongjia, Hubei, China

Forest type	ST (°C)		SWC (%)	
	Control	Trenched	Control	Trenched
EBF	13.23 ± 4.46^a^	13.74 ± 4.34^a^	22.74 ± 2.88^ab^	23.25 ± 1.68^bc^
MF	9.56 ± 3.17^b^	9.67 ± 3.21^b^	24.49 ± 4.13^a^	25.53 ± 3.58^a^
DBF	9.01 ± 2.98^b^	9.00 ± 2.63^b^	24.62 ± 4.10^a^	24.85 ± 4.41^ab^
CF	5.37 ± 2.33^c^	5.23 ± 2.01^c^	21.45 ± 1.92^b^	22.36 ± 1.81^c^

Note

Value was mean ± *SE*. Different letters indicated significant differences among different forest types (*p* < .05).

## 4 DISCUSSION

We presented findings from two years of soil CO_2_ fluxes of four montane forest types along an elevation gradient in northern China. We partitioned soil respiration fluxes into heterotrophic, autotrophic, and total soil respiration, and partitioned the variation of soil respiration resulted from the soil temperature, soil moisture, and the intersection of soil temperature and soil moisture. We found that soil temperature explained most of the soil respiration variation for four types of forest.

### 4.1 Soil respiration

Soil respiration rates of the four types of forest exhibited great seasonal variations along the elevation in Shennongjia, Hubei (Figure 2). Annual soil respiration efflux in EBF, MF, and DBF were 1.63 ± 0.06, 1.79 ± 0.06, and 1.72 ± 0.10 μmol CO_2_·m^–2^ s^–1^. This value was higher than those of temperate forest in northeastern China (1.07–1.36 μmol CO_2_·m^–2^ s^–1^; Wang, Dalal, Moody, & Smith, 2003) and in Thompson, MB, Canada (0.23–1.46 μmol CO_2_·m^–2^ s^–1^; Bond‐Lamberty, Wang, & Gower, 2004), but lower than those in tropical forests in Manaus, Brazil (4.36 μmol CO_2_·m^–2^ s^–1^; Malhi, Baldocchi, & Jarvis, 1999) and in Ouidah, Benin (4.25 μmol CO_2_·m^–2^ s^–1^; Lamade, Djegui, & Leterme, 1996). The annual soil respiration efflux in CF (1.35 ± 0.05 μmol CO_2_·m^–2^ s^–1^) was significantly lower than that in EBF, MF, and DBF (Table 3; *p* < .05). Compared with the broad‐leaved forests, the coniferous forest has a lower biomass, lower soil C storage, lower litter production, and a simpler community structure (Bréchet, Ponton, & Roy, 2009). Liu et al. (2012) explored the nutrient return of litter in deciduous broad‐leaved forests and evergreen coniferous forests in Shennongjia, and found that total nutrient return of litter of the deciduous broad‐leaved forest (303.3 kg hm^−2^ a^−1^) was significantly higher than that of the coniferous forest (244.0 kg hm^−2^·a^−1^). Deng et al. (2018) estimated forest carbon density of EBF, MF, DBF, and CF, and found that aboveground carbon in DBF (145.9 t C·ha^−1^) were significant higher than that in CF (128.0 t C·ha^−1^; Table 3). This indicated that the broad‐leaved forests had higher concentration of substrate and higher microbial activity, which lead to a higher soil respiration rate (Wang et al, 2003).

### 4.2 Contribution of heterotrophic respiration and autotrophic respiration to soil respiration efflux

We partitioned soil respiration fluxes into heterotrophic respiration and autotrophic respiration using the root‐cutting treatments. The annual soil CO_2_ efflux of autotrophic respiration in four types of forest along elevation in Shennongjia accounted for 31% (EBF), 38% (MF), 32% (DBF), and 40% (CF) of the total annual soil respiration efflux, respectively. Schlesinger (1997) found that the root respiration accounted for 30%–70% of the total soil respiration, and the studies in tropical forest had also demonstrated that the proportion of root respiration in total soil respiration was higher than 40% (Lamade et al., 1996 and Malhi et al., 1999), suggesting that the contribution of root respiration was relatively low in subtropical forests in Shennongjia. Bowden, Nadelhoffer, and Boone (1993) and Lee et al. (2003) found that root biomass was the main factor controlling the root respiration efflux, the autotrophic respiration. Thus the lower proportion of root respiration in total soil respiration might be the result of a lower root biomass in these forests (~2 Mg C ha^−1^, Table 7).

**Table 7:** Root biomass and root decay rate (*k*) in different root diameter classes in four types of forest along the elevation gradient in Shennongjia, Hubei, China

Forest type	Root biomass (g/m^2^)		Root decay rate (*k*; year^−1^)		*R* ^2^	
	*d* < 2 mm	*d* ≥ 2 mm	*d* < 2 mm	*d* ≥ 2 mm	*d* < 2 mm	*d* ≥ 2 mm
EBF	334.8 ± 11.9	1,670.8 ± 87.9	0.86	0.37	0.93	0.89
MF	516.9 ± 16.7	2,205.0 ± 90.7	0.81	0.29	0.98	0.83
DBF	711.0 ± 21.0	2,151.0 ± 102.1	0.86	0.37	0.85	0.91
CF	151.8 ± 6.98	1,501.4 ± 97.2	0.70	0.20	0.96	0.88

Note

Value was mean ± *SE*.

Abbreviation: *d*, diameter.

The contribution of autotrophic respiration was likely be underestimated, because the decomposition of severed roots may increase the measured soil respiration rates of deep collars (Díaz‐Pinés et al., 2010; Hanson et al., 2000; Kuzyakov, 2006; Subke et al., 2006). We found that the CO_2_ efflux from the severed roots in trenched plots was 14.6%–25.4% of the total soil respiration (Table 3). Lee et al. (2003) found that R_H_ in trenched plots was overestimated with 14%–52% due to the released CO_2_ efflux from the decomposition of the remaining fine roots (Bond‐Lamberty et al., 2004; Lee et al., 2003).

The soil heterotrophic respiration in four types of forest was the main contributor of soil respiration, which represented 69%, 62%, 68%, and 60% of soil respiration for EBF, MF, DBF, and CF along the elevation gradient in Shennongjia, respectively (Figure 2). Although the soil organic C and nitrogen content was different among the four type of forests, there was no significant difference in *R*_H_ for the broad‐leaved forests (Table 2). But *R*_H_ for the subalpine coniferous forests was significantly lower than the broad‐leaved forests. Adachi, Bekku, and Wan (2006) found a spatial heterogeneity of soil respiration in tropical mountain rain forest, which was influenced by other soil properties (Nottingham, Turner, & Chamberlain, 2012). This suggested that the mineral soil respiration was not only influenced by soil organic C and nitrogen content, but also affected by several other factors, such as availability of nutrients in the leaf litter, fine root biomass, and aboveground biomass (Campbell & Law, 2005; Ryan & Law, 2005). Epron, Nouvellon, and Roupsard (2004) indicated that soil respiration rate was not affected by soil organic C concentration, but by the forest litter production in tropical forest in Congo. So, we deduced that the heterotrophic respiration of the four types of forest in Shennongjia was mainly derived from the litter layer decomposition.

### 4.3 Responses of soil respiration to soil temperature and moisture

Soil temperature and soil moisture have been identified as the main drivers of the variation in soil respiration (Schimel et al., 1994; Wu, Zhang, Wang, Sun, & Guan, 2010; Zhou, Wan, & Luo, 2007). Temperature explained most of the variance in soil CO_2_ efflux in temperate forests, for example, 75%–90% in Minnesota forest (Reiners, 1968), 88% in Australian forest (Richards, 1981), 81% in London Clay forest (Anderson, 1973), 94% in Tennessee mixed deciduous forest (Edwards, 1975), 90%–96% in Japan forest (Nakane, Yamamoto, & Tsubota 1983), and 75%–89% in Florida pine plantation (Ewel, Cropper, & Gholz 1987). In Shennongjia Mountain, soil temperature explained 84.1%–89.7% of the variance of soil CO_2_ efflux (Table 5).

The response of soil CO_2_ efflux to the increase of temperature can be described by Q_10_ which is the temperature coefficient of the reaction. The Q_10_ value of soil respiration against soil temperature in the four types of forest in Shennongjia was 3.06 (EBF), 3.75 (MF), 4.05 (DBF), and 4.49 (CF; Table 4). The *Q*_10_ value of global forest soil respiration was about 2.4, and the *Q*_10_ value of soil respiration was higher at low temperature than at high temperature (Raich & Schlesinger, 1992). The previous studies on in tropical and subtropical forests suggested a range of 2.2 (1.4–4.6) of the *Q*_10_ of soil respiration (Chen & Tian, 2005), while *Q*_10_ value of soil respiration in temperate zones was 5.4 (Han & Jin, 2018). Compared with CF soil respiration, the *Q*_10_ value of EBF was lower, because EBF characterized with adequate supply of soil organic substrate, higher microbial activity, and more complex species composition than CF. The CF was located at a high altitude (2,570 m) in the Shennongjia Mountain, implying that the C storage in this forest was likely to suffer more disturbance under global warming because the *Q*_10_ of soil respiration in a high‐altitude forest was higher than that in a low‐altitude tropical forest (Zimmermann, Davies, & Peña de Zimmermann, 2015).

It was reported that the temperature sensitivity of the organic matter decomposition in soil was 2.3–4.9 (Zimmermann, Leifeld, & Conen, 2012). Harvard forest showed a decreased *Q*_10_ value after removal of root (Boone et al., 1998). In the present study, we found that *Q*_10_ increased significantly after removal of the root (Table 5). This suggested that temperature sensitivity of root respiration differed from that of the total soil respiration, and the root respiration had a lower temperature sensitivity.

Variation in soil temperature can account for most of the seasonal and diel variation in soil CO_2_ efflux, but the temperature effect was not always consistent, and other factors such as soil water content influenced soil respiration (Davidson et al., 1998). The relationship between moisture content and soil respiration varied temporally depending on the stage of soil wetting and drying cycles (Keith, Jacobsen, & Raison, 1997). Rapid declines in soil respiration in respond to soil water saturation had been observed in seasonal forest in the Amazon (Sotta, Meir, Malhi, Nobre, & Hodnett, 2004) and in moist tropical forest in Panama (Kursar, 1989). The decline in soil respiration in respond to increased soil moisture could be the result of reduced diffusion of CO_2_ from saturated soils (Schwendenmann & Veldkamp, 2006). Reduced soil CO_2_ efflux could also be due to reduced soil microbial activity in low O_2_ environments (Orchard & Cook, 1983). In Shennongjia Mountain, soil moisture sensitivity of soil autotrophic respiration was significantly different among the four types of forests, and soil temperature explained 62%–81% of variation in respiration (Table 5). Combined with the soil moisture, soil temperature and soil moisture together explained 91%–97% of soil respiration variation for the four types of forests (Table 5). Moisture in soils was essential for both plant growth and soil microbial activity, thus affecting carbon inputs as well as the decomposition of litter and soil organic matter, and hence heterotrophic respiration and carbon outputs (Moyano et al., 2013). The results indicated that integrating soil moisture into soil respiration–temperature models improved the robustness of the prediction of soil respiration (Davidson et al., 1998; Law et al., 2001; Raich & Schlesinger, 1992; Tang & Baldocchi, 2005).

Soil respiration was highly sensitive to soil temperature and soil moisture, and the intersection effects of soil temperature and soil moisture on soil respiration were complex (Schlesinger, 1977). Schlentner and van Cleve (1985) found that the effect of one variable on soil respiration depended on the range of the other variable. Carlyle and Than (1988) found that soil temperature had no effect on soil respiration when the soil moisture was below a critical content. Some previous researches had focused on the effect of either soil temperature or soil moisture on soil respiration. Lots of studies have found that soil temperature explained 75%–90% of the variance in soil respiration (Keith et al., 1997), and soil temperature and soil moisture together explained over 90% of the variance in soil respiration of six temperate forest (Wang et al., 2003). In this study, we parameterized the relationship by linear and exponent regression model and quantified the intersection effects between temperature and moisture on soil respiration. We found that the intersection effects of soil temperature and soil moisture explained 10.9%–25.0% of variation in soil respiration and 17.1%–19.6% of variation in soil autotrophic respiration (Table 5). Our analysis clearly demonstrated that the intersection effects of soil temperature and soil moisture on soil respiration are essential to understand the mechanism of climate controls on both soil respiration and its components.

## 5 CONCLUSION

Numerous studies have reported that both soil temperature and soil moisture are major drivers of soil respiration in forest ecosystems. But few field studies have quantified the intersection of these two factors on soil respiration and its components. Here, we quantified the relative contribution of soil temperature, soil moisture, and their intersection on the variation of soil respiration and its two components of four types of forest along a natural elevation gradient in Shennongjia, Hubei, China. We found that the intersection effects between soil temperature and soil moisture accounted for 17.1%–19.6% of variation in *R*_A,_ but only 1.0%–7.0% of variation in *R*_H_, respectively. However, the proportion of variation in *R*_H_ explained by the intersection increased with elevation. Up to now, the mechanism of how soil temperature and moisture determined soil respiration and its two components remains unclear. Thus, a clear understanding of forest soil respiration and its driving forces, especially the intersection driving effect of soil temperature and soil moisture, is an essential step toward predicting effects of climate change and formulating policy on forest carbon management.

## CONFLICT OF INTEREST

None declared.

## AUTHORS CONTRIBUTION

Guozhen Shen conceived the ideas and designed the research; Mingzhe Ma, Guozhen Shen, and Zhenhua Zang collected data; Mingzhe Ma, Zhenhua Zang, Guozhen Shen, and Zongqiang Xie analyzed data and wrote the manuscript; All authors contributed critically to the drafts and gave final approval for publication.

## Data Availability

The raw data upload as Appendix S1, see the file: rawdata.zip.
